# Two UGT84A Family Glycosyltransferases Regulate Phenol, Flavonoid, and Tannin Metabolism in *Juglans regia* (English Walnut)

**DOI:** 10.3389/fpls.2021.626483

**Published:** 2021-02-24

**Authors:** Houston J. Saxe, Takanori Horibe, Bipin Balan, Timothy S. Butterfield, Noah G. Feinberg, Christopher M. Zabaneh, Aaron E. Jacobson, Abhaya M. Dandekar

**Affiliations:** ^1^Department of Plant Sciences, University of California, Davis, Davis, CA, United States; ^2^College of Bioscience and Biotechnology, Chubu University, Kasugai, Japan; ^3^ARS Crops Pathology and Genetics Unit, United States Department of Agriculture, Davis, CA, United States

**Keywords:** glucosyltransferase, hydrolysable tannin, UGT84A, gallic acid, walnut, phenol metabolism, transcriptomics, metabolomics

## Abstract

We showed previously that gallic acid is produced in walnut from 3-dehydroshikimate by a shikimate dehydrogenase (JrSkDH). This study focuses on the next step in the hydrolysable tannin pathway, the formation of 1-*O*-galloyl-β-D-glucose from the phenolic gallic acid and UDP glucose by a glycosyltransferase. JrGGT1 (UGT84A73) and JrGGT2 (UGT84A74) are predicted to be two such glycosyltransferases, which we expressed in tobacco plants. GC-MS analysis of the transgenic tobacco revealed moderate, yet significant alterations in plant secondary metabolism, such as depleted phenolic acids, including gallic acid. We postulate that these effects are due to JrGGT1 and JrGGT2 activity, as JrGGT orthologs glycosylate these phenolic compounds *in vitro*. Moreover, *JrGGT* expression in tobacco caused upregulation of shikimic acid pathway metabolites and differing responses in phenylpropanoids, such as phenolic acids and flavonoids. In transcriptome analysis of walnut pellicle tissues, both *JrGGT*s showed substantial and significant expression correlations with the gallic acid-producing *JrSkDH*s and were highly coexpressed with the genetic circuits constituting the shikimic acid and phenylpropanoid biosynthetic pathways. Verification of *JrGGT* gene expression by transcriptome analysis of 20 walnut tissues revealed striking similarities with that of the pellicle data, with the greatest expression in roots, wood, buds, and leaves of *Juglans regia* cv. Chandler: tissues that typically accumulate hydrolysable tannins. Like the transgenic tobacco, pellicle metabolomic analyses revealed that many phenylpropanoids correlated negatively with *JrGGT* expression, while shikimic acid pathway metabolites correlated positively with *JrGGT* expression. This research supports the hypothesis that JrGGT1 and JrGGT2 play non-trivial roles in metabolism of phenolic acids, flavonoids, and ostensibly, tannins.

## Introduction

Phenolic compounds are a large class of plant secondary metabolites that include phenolic acids, flavonoids, and tannins (Abbas et al., 2017). Phenolic acids and flavonoids are considered “simple” compared to the more “complex” tannins, since the latter are usually polymers of the former. These phytochemicals help plants endure stresses due to water, heat, physical damage, pathogens, and herbivory (Sharma et al., 2019). Phenolic compounds are bioactive, highly bioavailable, and beneficial to human health, protecting against the adverse effects of hyperlipidemia and hyperglycemia and showing anticancer activities (Khan and Mukhtar, 2019).

**FIGURE 1:**
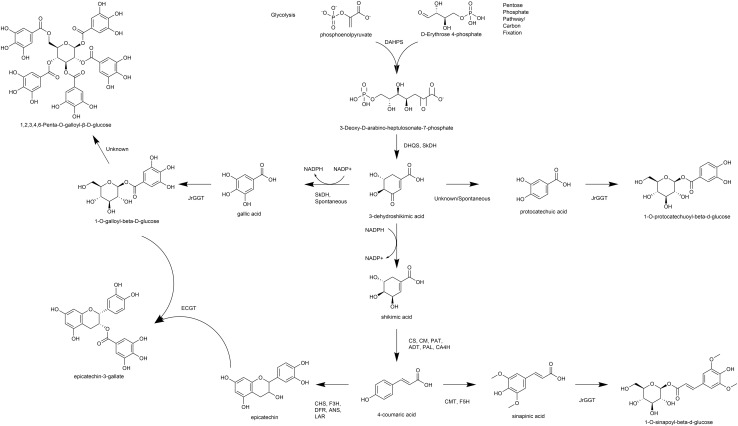
Overview of pathways, enzymes, and metabolites primarily associated with HBA/HCA UGT activity. DAHPS, 3-deoxy-D-arabinoheptulosonate 7-phosphate synthase; DHQS, dehydroquinate synthase; SkDH, bifunctional dehydroquinate dehydratase/shikimate dehydrogenase; CS, chorismate synthase; CM, chorismate mutase; PAT, bifunctional aspartate aminotransferase and glutamate/aspartate-prephenate aminotransferase; ADT, arogenate dehydratase/prephenate dehydratase; PAL, phenylalanine ammonia lyase; CA4H, cinnamic acid-4-hydroxylase; CMT, caffeic acid 3-*O*-methyltransferase; F5H, ferulate-5-hydroxylase; CHS, chalcone synthase; F3H, naringenin, 2-oxoglutarate 3-dioxygenase; DFR, dihydroflavonol-4-reductase; LAR, leucoanthocyanidin reductase; ANS, leucoanthocyanidin dioxygenase; UGT, uridine diphosphate glycosyltransferase.

Biosynthesis of phenylpropanoids like phenolic acids and flavonoids begins at the shikimic acid trunk. The irreversible condensation of erythrose-4-phosphate (E-4-P) from the pentose phosphate pathway and phosphoenolpyruvate (PEP) from glycolysis by 3-deoxy-D-arabinoheptulosonate 7-phosphate synthase (DAHPS) forms the seven-carbon 3-deoxy-D-arabinoheptulosonate 7-phosphate (Widhalm and Dudareva, 2015) (Figure 1). Cyclization of deoxyarabinoheptulosonate by a dehydroquinate synthase (DHQS) results in the formation of 3-dehydroquinate (3-DHQ). Catalysis of the third and fourth reactions of the shikimic acid trunk in *J. regia* occurs by a bifunctional shikimate dehydrogenase (JrSkDH) (Muir et al., 2011) (Figure 1). JrSkDH facilitates a two-step reaction in which 3-DHQ is converted to 3-dehydroshikimate (3-DHS) by the dehydratase subunit and is subsequently acted on by the NADP+/(H)-dependent dehydrogenase subunit to produce shikimate (Muir et al., 2011). This assembly of two enzymes is different than in bacteria, where dehydratase and dehydrogenase are separate enzymes (Michel et al., 2003; Muir et al., 2011; Maeda and Dudareva, 2012).

The first committed step of phenylpropanoid metabolism begins past the shikimic acid trunk and encompasses the biosynthesis of hydroxycinnamic acids (HCA) and flavonoids. Phenylalanine ammonia-lyase (PAL) deaminates phenylalanine to produce ammonia and the HCA cinnamic acid (Blount et al., 2000). Cinnamic acid is then hydroxylated to produce 4-HCA (also p-coumaric acid) by cinnamic acid 4-hydroxylase (C4H), which is followed by 4-coumarate-CoA ligase (4CL) to produce *p*-coumaroyl CoA (Deng and Lu, 2017). At this step, *p*-coumaroyl CoA can commit either to the flavonoid pathway via chalcone synthase (CHS) or to the HCA pathway via hydroxycinnamoyl-CoA: shikimate/quinate hydroxycinnamoyl transferase (HCT) (Deng and Lu, 2017). HCT acts on *p*-coumaroyl CoA to produce caffeoyl shikimic acid and caffeoyl quinic acid (QA; chlorogenic acid). Caffeoyl shikimic acid can then be converted to caffeic acid by coumaroylshikimate esterase (CSE). Alternatively, 4-HCA can be converted to caffeic acid directly by C4H (Chen et al., 2011). Ferulic and sinapinic are biosynthesized successively by CMT with the methyl group being incorporated by S-adenosylmethionine (Urban et al., 1994; Wang et al., 2018, 2019). CHS constitutes the first committed step of flavonoid biosynthesis by condensation of 4-coumaroyl-CoA (from 4-HCA) and malonyl-CoA and thus is linked tightly to phenylpropanoid metabolism (Nakayama et al., 2019) (Figure 1). Subsequently, the flavanones, including naringenin, are formed by chalcone isomerase (CHI), which are acted on by flavanone 3-hydroxylase (F3H) to produce the flavanols. Dihydroflavonol-4-reductase (DFR) acts on the flavonols to make the leucoanthocyanidins, which can be channeled to the flavan-3-ols by leucoanthocyanidin reductase (LAR) or to the anthocyanidins by anthocyanidin synthase (ANS) (Nakayama et al., 2019).

The polyphenolic hydrolysable tannins (HTs) consist of a central glucose moiety that is esterified with at least five galloyl residues (Grundhöfer et al., 2001) (Figure 1). The HTs constitute a class of polyphenolic compounds found abundantly in the oak family that includes walnut (Order Fagales). HTs are used for tanning leather because of their unique property to precipitate proteins, which may also be related to their ability to provide pest, pathogen, and metal toxicity resistance.

We were the first to show that gallic acid (GA), a key intermediate in the biosynthesis of HTs, is made enzymatically from a shikimate dehydrogenase (SkDH) via the shikimic acid pathway (Muir et al., 2011) (Figure 1). This enzyme represents a branching point in the pathway, where 3-DHS can either be oxidized to the hydroxybenzoic acid (HBA) GA (NADP+-dependent), spontaneously converted to the HBA GA and PCA, or be reduced to shikimic acid (NADPH-dependent) (Muir et al., 2011; Bontpart et al., 2016) (Figure 1).

The first committed step in HT biosynthesis is formation of 1-*O*-galloyl-β-D-glucose from GA and UDP-glucose (UDPG) by a UDP-glycosyltransferase (UGT) (Gross, 2008) (Figure 1). Experiments in oak (*Quercus robur*), pomegranate (*Punica granatum*), grape (*Vitis vinifera*), strawberry (*Fragaria × ananassa*), and tea (*Camellia sinensis*) have characterized genes and enzymes with UGT activity toward phenolic acids and flavonoids, with particular emphasis on GA (Lunkenbein et al., 2006; Khater et al., 2012; Mittasch et al., 2014; Cui et al., 2016; Ono et al., 2016). Double RNAi knockdown of UGT84A23 and UGT84A24 in pomegranate hairy roots drastically reduced HTs in these tissues (Ono et al., 2016). Moreover, biosynthesis of the famous tea tannins, epicatechin gallate, and epigallocatechin gallate was achieved with 1-*O*-galloyl-β-D-glucose acting as the galloyl donor (Liu et al., 2012). Interestingly, the *JrGGT* orthologs display different degrees of promiscuity among phenolic compounds. For example, UGT84A13 from oak displayed a clear preference for glycosylating HBAs over HCAs by specific activity (Mittasch et al., 2014). Conversely, UGT84A23 and UGT84A24 from pomegranate did not discriminate between HBAs and HCAs and even showed activity with some flavonoids (Ono et al., 2016). Similarly, UGT84A22 from tea did not prefer HBAs over HCAs (Cui et al., 2016). Lastly, UGT84A44 (VvgGT1), VvgGT2, and VvgGT3 displayed an insignificant, slight inclination for HBAs over HCAs, with no significant activity detected with flavonoids (Khater et al., 2012).

This class of glycosyltransferases (GTs) is grouped into the 1-glycosyltransferase family (of 108 families) and relies upon uridine diphosphate (UDP) sugars as the donor substrate, according to the Carbohydrate-Active enZYmes Database (CAZy) (Lombard et al., 2014). The 1-glycosyltransferases are further organized into 16 groups (A-P) (Caputi et al., 2012). Using the group L UGT from *Quercus robur* UGT84A13 (Mittasch et al., 2014) and the UGT conserved PSPG motif, the *Juglans regia* transcriptome was queried and ∼ 130 different UGT genes were identified (Martinez-Garcia et al., 2016). In a search for enzymes with putative UGT84A activity, two genes shared the greatest similarity to the group L UGT84A13 and were named *JrGGT1* and *JrGGT2*. While many studies have elucidated *in vitro* functions of UGT84As, few have examined their *in vivo* effects and behavior as we present in this paper (Lunkenbein et al., 2006; Khater et al., 2012; Mittasch et al., 2014; Cui et al., 2016; Ono et al., 2016). The genomics, transcriptomics, and resultant metabolomics of the putative tannin biosynthetic enzymes are presented in this study. We intend to build upon the current body of knowledge to elucidate the genetic and metabolic background of the first committed step of HT biosynthesis in *Juglans regia*.

## Materials and Methods

### *J. regia* Somatic Embryos and *N. tabacum* SR1 Plants

*JrGGT1* and *JrGGT2* sequences were amplified from DNA extracted from somatic embryo tissue of *Juglans regia* cv. Chandler. *Nicotiana tabacum* cv. Petit Havana (SR1 tobacco) was used in transformation experiments to express *JrGGT1* & *2*.

### *J. regia* Pellicle Tissue

Three cultivars of *Juglans regia* were chosen for transcriptome and metabolome profiling. Walnuts were collected in the UC Davis campus research orchards. A single tree of each cultivar was identified and tagged for repeated nut harvests. Nuts were harvested from each at three developmental stages: Stage 2 (85 days post-peak bloom), Stage 3 (110 days post-peak bloom), and Stage 4 (150 days post-peak bloom). At each harvest, nuts were chosen from the lower perimeter of the tree in each cardinal direction. Nuts of similar proportions representing five biological replicates were removed, placed in a one-gallon Ziploc© bag, and transported back to the laboratory on wet ice in a cooler. Nut length and width were measured with a caliper to ensure consistent maturity within each sample set. The nuts were dissected quickly to remove pellicle tissues; excised pellicle tissue was frozen in liquid N2 and kept frozen at −80°C. Frozen, excised pellicles were ground under liquid N2, and 0.10 to 0.15 g of powdered tissue was used for transcriptome and metabolome analysis.

### Gene Characterization

*JrGGT1* and *JrGGT2* sequences were identified previously (Martinez-Garcia et al., 2016). BLASTP (a trademark of NCBI, Basic Local Alignment Search Tool) was also performed using the NCBI-predicted, full-length amino acid sequence of *JrGGT1* as a query. In addition to *JrGGT2*, seven functionally characterized UGTs and one non-functionally characterized UGT were selected from these results.

### Cloning of *JrGGT1* and *JrGGT2* and Expression in Tobacco

Genomic DNA was extracted from somatic embryos of *Juglans regia* cv. Chandler using NucleoSpin Plant II (TaKaRa Bio, Japan). Four primer sets were designed according to the *Juglans regia* genome database in NCBI to clone *JrGGT*s (*JrGGT1* and *JrGGT2*) from walnut (Supplementary Table 1). PCR reactions were conducted using genomic DNA as a template. Primers “FW: *JrGGT1*” and “Rv: *JrGGT1*” were used to clone *JrGGT1*, while “FW: *JrGGT2*” and “Rv: *JrGGT2*” were used to clone *JrGGT2*. PCR reactions were as follows: one cycle of 98°C for 1 min followed by 36 cycles of 98°C for 10 s, 60°C for 15 s, and 68°C for 90 s. To avoid carryover of template DNA in the cloning reactions, all PCR fragments were purified by 0.8% (w/v) agarose gel electrophoresis and subsequent spin column recovery using NucleoSpin Gel and PCR Clean-up (TaKaRa Bio, Japan). The amplicons were subsequently cloned into the pCR2.1-TOPO vector using a TOPO TA Cloning Kit (Thermo Fisher Scientific, United States). The resultant plasmids were transformed into *E. coli* by heat shock using TOP10 competent cells (Thermo Fisher Scientific, United States). Colonies containing each plasmid (*JrGGT1* and *JrGGT2*) were screened by PCR using primers “FW: *JrGGT1*,” “Rv: *JrGGT1*,” “FW: *JrGGT2*,” and “Rv: *JrGGT2*.” Plasmid DNA from selected colonies was isolated (QIAprep Spin Miniprep Kit, Qiagen, Germany) and sequenced (Quintara Biosciences, United States).

After confirmation of native *JrGGT* sequences, we synthesized *JrGGT1* and *JrGGT2* coding sequences (CDS) to include an encoded six-histidine tag at the 3′ end (Supplementary Figure 1). These synthesized fragments were inserted into a binary vector with In-Fusion HD Cloning (Clontech, United States) to generate *JrGGT1* and *JrGGT2* overexpressing vectors (Supplementary Figure 1). The resultant plasmids, pDH17.0301 (*JrGGT1*) and pDH17.0401 (*JrGGT2*), were transformed into *E. coli* HST08 by heat shock. After proper selection, DNA was extracted from Stellar competent cells containing pDH17.0301 (*JrGGT1*) and pDH17.0401 (*JrGGT2*), then electroporated into *Agrobacterium tumefaciens* strain EHA 105 PCH32 according to a protocol adapted from a previously published one (Shen and Forde, 1989). Then, pDH17.0301 and pDH17.0401 were used to transform *N. tabacum cv.* SR1 using *Agrobacterium*-mediated plant transformation at the Ralph M. Parsons Plant Transformation Facility^1^.

### RNA Extraction of Pellicle Tissue and cDNA Library Preparation

Total mRNA was extracted from pellicles representing five independent biological replicates (each nut being a biological replicate) for each of three genotypes and three time stages for 45 samples total. The PureLink Plant RNA Reagent (Invitrogen; Carlsbad, CA, United States) was used for the extraction per manufacturer’s protocol, with modifications. Due to the low water content and high lipid concentration in pellicles, the ratio of PureLink Reagent: tissue was increased from 500 μL PureLink Reagent: 0.1 g tissue to 500 μL PureLink Reagent: 0.025 ground pellicle, a four-fold increase in the extraction buffer. RNA pellets were resuspended in 30 μL nuclease-free water (Ambion; Austin, TX, United States) and suspended overnight on wet ice in a 4°C chamber for 16 h. The construction of cDNA libraries was performed in accordance with a published protocol (Illumina TruSeq Stranded Sample Preparation Guide 2014). Total RNA molecules were chemically fragmented in the presence of Fragment, Prime Finish Buffer for 8 min at 94°C, followed by a 4°C incubation to arrest the chemical cleavage of mRNA molecules. These fragmentation patterns yield, on average, a cDNA insert size of 120 bp. A BIOO Scientific NEXTflex^TM^ Rapid Directional RNA-seq^TM^ Library Prep Kit Adapter kit containing 48 unique adapter sequences was used to generate uniquely labeled samples. Thirty microliters of uniquely barcoded cDNA libraries were transferred to non-stick, RNAse-free 0.5 mL microfuge tubes (Ambion, Austin, TX, United States) and stored at −20°C prior to sequencing by synthesis.

### Sequencing by Synthesis—RNA-Seq and Bioinformatics Analysis of RNA-Seq Data

cDNA sequencing was conducted at the UC Davis Expression Analysis Core Facility (UCD EACF) using an Illumina HiSeq 3000 machine. Each cDNA library was sequenced in one direction for 50 nucleotides (NT). The quality of the raw reads was checked using FastQC^2^, and the low-quality bases were trimmed using a custom-made Perl script. The adapters were removed using Cutadapt Version 1.8.1 (Martin, 2011). To remove the rRNAs, the cleaned reads were aligned to the SILVA rRNA database^3^ using Bowtie Version 2.2.8 (Langmead et al., 2009) and only the unaligned reads were considered for further analysis. The preprocessed reads were then mapped to the walnut (*Juglans regia*) reference genome downloaded from NCBI^4^ using “splicing-aware” aligner HISAT (hierarchical indexing for spliced alignment of transcripts; Version 2.0.5) (Kim et al., 2015). The normalized gene expression levels in FPKM (fragments/kb transcript per million mapped reads) of all samples were estimated using Cufflinks Version 2.2.1 (Trapnell et al., 2013). The RNA-Seq data analysis workflow is illustrated in Supplementary Figure 2.

### Purification of Recombinant Protein

The protein was extracted by weighing 400 mg frozen leaf tissue into 2-mL microcentrifuge tubes. A 3-mm stainless steel bead was then added to each tube along with 1 mL binding buffer (Milli-Q water, 50 mM Tris–HCl, 20 mM imidazole, 500 mM NaCl, and 15% (v/v) glycerol; pH adjusted to 7.6). The samples were lysed in a Qiagen Retsch MM300 TISSUELYSER (Haan, Germany) at maximum frequency for 90 s and subsequently centrifuged at 20,000 × *g* for 20 min. The supernatant was then purified using the Zymo His-Spin Protein Miniprep kit (Irvine, CA, United States). The purified eluted protein was then added to an Amicon Ultra-0.5 Centrifugal Filter Unit (30 kDa) and centrifuged at 14,500 × *g* until 40 μL of volume was achieved. Then, 450 μL dilution buffer (Milli-Q water, 50 mM Tris–HCl and 20% (v/v) glycerol; pH 7.6) was added to 40 μL protein (total volume 490 μL) and centrifuged until 40 μL was achieved for a second time. This step was repeated twice to remove most of the imidazole and NaCl and to concentrate the protein. The protein was then quantified using Qubit Fluorometric Quantification (Thermo Scientific, Waltham, MA, United States).

### Metabolomic Analysis

#### *JrGGT1*- and *JrGGT2*-Overexpressing Tobacco

Leaf tissue samples representing six independent biological replicates, each from *JrGGT1*- and *JrGGT2*-overexpressing tobacco lines, and leaf tissue samples representing seven independent biological replicates from UT lines were sent to the West Coast Metabolomics Center (WCMC) in the Genome Center at UC Davis for primary metabolism analysis by gas chromatography/time-of-flight mass spectrometry (GCTOF). Twenty ±5-mg fresh weight of tobacco leaves were harvested and frozen immediately in liquid nitrogen. Tissue was homogenized under liquid nitrogen using a Retsch mill. Two mL of a single-phase solvent mixture of methanol/chloroform/water 2.5:1:1 v/v/v kept at −20°C was added to the tissue and thoroughly mixed at 4°C for 30 min to precipitate proteins and DNA/RNA and to disassociate metabolites from membrane and cell wall components. After centrifugation, the remaining pellet consisting of DNA/RNA, proteins, starch, membranes, and cell wall components was extracted in a second step with 1 mL methanol/chloroform 1:1 v/v at −20°C. The organic solvent extracts were combined and used for metabolite analysis via GC-TOF.

For GC-TOF MS (Leco Pegasus IV GC-TOF mass spectrometer; Leco, St. Joseph, MO, United States) analysis, the organic phase was dried and dissolved in 50 mL methoxamine hydrochloride (20 mg/mL pyridine) and incubated at 30°C for 90 min with continuous shaking. Then, 80 mL *N*-methyl-*N*-trimethylsilyl trifluoroacetamide (MSTFA) was added at 37°C for 30 min to derivatize polar functional groups. The derivatized samples were stored at room temperature for 120 min before injection. GC-TOF analysis was performed on an HP 5890 gas chromatograph with tapered, deactivated split/splitless liners containing glass wool (Agilent, Böblingen, Germany) and 1 mL split-less injection at 230°C injector temperature. The GC was operated at a constant flow of one mL/min helium with a 40 m 0.25 mm id 0.25 mm RTX-5 column with 10 m integrated precolumn. The temperature gradient started at 80°C, was held isocratic for 2 min, and subsequently ramped at 15°C/min to a final temperature of 330°C, which was held for 6 min. Twenty spectra per second were recorded between m/z 85 and 500. Peak identification and quantification were performed using the Pegasus software package (Leco, St. Joseph, MO, United States).

Reference chromatograms were defined that had a maximum of detected peaks over a signal/noise threshold of 20 and were used for automated peak identification based on mass spectral comparison to a standard NIST98 library (Mclafferty et al., 1999). Automated assignments of unique fragment ions for each individual metabolite were taken as default as quantifiers and manually corrected where necessary. All artifactual peaks caused by column bleeding or phthalates and polysiloxanes derived from MSTFA hydrolyzation were identified manually and removed from the results table.

Metabolite data were then normalized using the mTIC method by calculating the sum of all peak heights for all identified metabolites (but not the unknowns) in each sample. Such peak sums are called “mTIC” in analogy to the term TIC used in mass spectrometry (for “total ion chromatogram”), but with the notification “mTIC” to indicate that only genuine metabolites (identified compounds) were used to avoid using potential non-biological artifacts for the biological normalizations, such as column bleed, plasticizers, or other contaminants.

Subsequently, it was determined whether the mTIC averages are significantly different between treatment groups or cohorts. If these averages were different by *p* < 0.05, data was normalized to the average mTIC of each group. If averages between treatment groups were not different, data was normalized to the total average mTIC.

#### Metabolite Analysis of *J. regia* Pellicle Tissue

Pellicles representing five biological replicates (each nut being a biological replicate) for each of the three genotypes at each of the three time stages, for a total of 45 samples, were shipped to Metabolon (Research Triangle Park, NC, United States) for sample preparation and analysis. Pellicle samples were prepared using the automated MicroLab STAR^®^ system from Hamilton Company (Reno, NV, United States) with the addition of recovery standards. To disassociate protein-bound metabolites, the samples were mixed with methanol and shaken vigorously for 2 min using a Glen Mills GenoGrinder 2000 (SPEX Sample Prep; Metuchen, NJ, United States); proteins were pelleted by centrifugation. Sample extracts were dried, then reconstituted in solvents compatible with four methods: two for analysis on two separate reversed-phase (RP) ultraperformance liquid chromatography (UPLC)–mass spectroscopy (MS/MS) with positive ion electrospray ionization (ESI) platforms; one for analysis on an RP/UPLC-MS/MS with negative ESI platform; and one for analysis by hydrophilic interaction chromatography (HILIC)/UPLC-MS/MS with negative ion mode ESI. One sample was reserved for backup. Each solvent contained a series of standards at fixed concentrations to ensure injection and chromatographic consistency.

A global, unbiased metabolic profiling of *J. regia* pellicles was performed using a Waters (Milford, MA, United States) ACQUITY ultra-performance liquid chromatography (UPLC) and a Thermo Scientific (Waltham, MA, United States) Q-Exactive high-resolution/accurate mass spectrometer interfaced with a heated electrospray ionization (HESI-II) source with an Orbitrap mass analyzer for metabolite separation and identification. The MS analysis alternated between MS and data-dependent MSn scans using dynamic exclusion. The scan range varied slightly between methods but covered 70 to 1000 m/z. Raw data files were archived and extracted as described below.

Compounds were identified by comparison to a library of >3300 purified, authenticated standards containing the retention time/index (RI), m/z, and chromatographic data (including MS/MS spectra) of each molecule. Identifications were based on three criteria: retention index, accurate mass match to the library, and a comparison of MS/MS forward and reverse scores. Artifactual peaks were identified and removed by comparing experimental samples with process (ultra-pure water) and solvent blanks. Peaks were quantified using area-under-the-curve. To correct for minor variations, overall process variance (which includes MS peak quantitation variance) was established by running a pool of technical replicates made from a pool of experimental replicates interspersed evenly among the experimental samples throughout each run day. Data normalization was performed to correct instrument inter-day tuning differences. Essentially, each compound was corrected in run-day blocks by registering the medians to equal one (1.00) and normalizing each data point proportionately.

### Free Amino Acid Analysis

#### Extraction

Free amino acids were extracted from tobacco leaf tissue samples representing six independent biological replicates each for *JrGGT1* and *JrGGT2* expressing and UT lines based as described (Hacham et al., 2002). One hundred and fifty milligrams of frozen tissue was weighed into two-mL Eppendorf tubes and cooled in liquid nitrogen. Six hundred microliters of water:chloroform:methanol (3:5:12 v/v) and a metal lysing bead were added to each tube. The samples were lysed in a Qiagen Retsch MM300 TISSUELYSER at maximum frequency for 90 s and sonicated in a Sonicor for 15 min. After sonication, the lysates were centrifuged at 21,000 × *g* for 10 min. The supernatants were collected, and the pellets were extracted once more. After combining supernatants, 300 μL chloroform and 450 μL water were added to each sample and centrifuged for 2 min. The resultant samples were separated into a chloroform phase at the bottom and water/methanol phase on top. The upper water/methanol phase was collected into new two-mL Eppendorf tubes and evaporated to dryness at room temperature using a speed-vac. The samples were then stored at −20°C. The Molecular Structure Facility at UC Davis conducted the analysis as described below.

#### Quantitative Analyses of Free Amino Acids in Physiological Fluids

Extracted sample pellets from *JrGGT1*- and *JrGGT2*-expressing and UT tobacco were dissolved in 100 nmol/mL AE-Cys (internal standard) Li (lithium) diluent before the 50-μL injection. Free amino acids were separated using strong cation-exchange chromatography with a post-column ninhydrin reaction for detection. Hitachi (Chiyoda, Tokyo, Japan) supplied column and buffers, and Wako (Richmond, Virginia) supplied ninhydrin. Calibration of the Hitachi 8900 was performed using amino acid standards (Sigma-Aldrich, St. Louis, MO, United States). Absorbance was recorded at both 570 nm and 440 nm after the reaction with ninhydrin to determine the response factor for each amino acid and to quantify relative to the known amino acid standards. The included internal standard (AE-Cys) was used to correct for any variance in injection volume due to the auto-sampler.

### Statistical Analysis

#### Feature Correlation Analysis of RNAs and Metabolites of *J. regia* Pellicle Tissue

RNA and metabolite data from pellicle tissue were analyzed using MetaboAnalystR (Xia et al., 2015). The “peak intensities” option was selected for data preprocessing. No data filtering was conducted. No normalization was conducted as Spearman correlation is a non-parametric analysis. MetaboAnalyst R function, FeatureCorrelation, was used with the argument, method = “spearman,” to return all RNAs and metabolites associated with *JrGGT1* and *JrGGT2* expression. The significance threshold was set at *P* < 0.05. Features meeting this threshold will be termed “significant” throughout this paper. Significance for metabolome pellicle (Mp) and transcriptome pellicle (Tp) were calculated using 45 independent biological replicates. Significance for T20 was calculated using 20 independent biological replicates.

#### Metabolomics Analysis of *JrGGT1* and *JrGGT2* Overexpressing Tobacco

The metabolomics data were analyzed using MetaboAnalystR. The “peak intensities” option was selected for data preprocessing, and no IQR filtering was performed. Normalization by auto-scaling and general logarithm transformation was used to achieve normal distributions. Principal component analysis was performed to observe unbiased sample clustering, while partial least-square discriminant analysis (PLS-DA) was used to determine differentially expressed metabolites. Metabolites were only considered “significant” or “differentially expressed” if they had a variable importance in projection (VIP) score of one or higher. Features meeting this threshold are termed “significant” throughout this paper.

#### Free Amino Acid Analysis of *JrGGT1*- and *JrGGT2*-Overexpressing Tobacco

The free amino acid data were analyzed using MetaboAnalystR. The “peak intensities” option was selected for data preprocessing and IQR filtering was performed. Normalization by auto-scaling and general logarithm transformation was used to achieve a more normal distribution. Partial least-square discriminant analysis (PLS-DA) was used to determine differentially expressed metabolites. Metabolites were only considered “significant” or “differentially expressed” if they had a variable importance in projection (VIP) score of one or higher. Features meeting this threshold are termed “significant” throughout this paper.

## Results

### Cloning of *JrGGTs*

In an earlier study, we identified two genes, *JrGGT1* and *JrGGT2*, based on their significant homology to *QrUGT84A13* and postulated that these two genes were likely candidates involved in the synthesis of 1-*O*-galloyl-β-D-glucose, an essential precursor for synthesis of hydrolysable tannins (HTs) (Martinez-Garcia et al., 2016). There was close structural similarity of *JrGGT1*, located on chromosome 16, and *JrGGT2*, present on chromosome 13, to enzymes from oak, pomegranate, grapevine, and tea (Table 1). In the present study, we performed a functional analysis of these two genes expressed in tobacco, combined with RNA-seq and metabolomics analyses of walnut tissues. Based on the recommendation of the UNC, *JrGGT1* was named UGT84A73 and *JrGGT2* was named UGT84A74 (Mackenzie et al., 2005). We cloned full-length protein-encoding regions corresponding to *JrGGT1* and *JrGGT2* directly from DNA extracted from *Juglans regia* cv. Chandler using the primers shown in Supplementary Table 1 since neither gene has introns. The coding sequences for both were contiguous in the walnut genome.

**TABLE 1:** BLASTP results for *JrGGT1*, including selected functionally characterized HBA/HCA UGTs.

**Description**	**Chromosome location**	**Max score**	**Total score**	**Query cover**	**E value**	**% ident**	**Accession**
*JrGGT1* (*J. regia*)	16	1058	1058	100%	0	100%	XP_018828935.2
*JrGGT2* (*J. regia*)	13	982	982	100%	0	91.78%	XP_018827666.1
QlGGT (*Q. lobata*)	11	938	938	100%	0	87.87%	XP_030943776.1
UGT84A13 (*Q. robur*)	?	934	934	100%	0	87.48%	V5LLZ9.1
UGT84A24 (*P. granatum*)	4	862	862	99%	0	80.85%	XP_031391018.1
UGT84A23 (*P. granatum*)	2	853	853	99%	0	79.38%	XP_031382117.1
VvgGT2 (*V. vinifera*)	?	848	848	93%	0	83.40%	AEW31188.1
VvgGT1 (*V. vinifera*)	?	845	845	93%	0	83.61%	AEW31187.1
UGT84A22 (*C. sinensis*)	?	843	843	93%	0	83.37%	ALO19890.1
FaGT2 (*Fragaria x ananassa*)	?	830	830	98%	0	77.98%	Q66PF4.1

### *JrGGT1* and *JrGGT2* Coexpressed With Genes and Metabolites Responsible for HBA, HCA, and Flavonoid Biosynthesis

**FIGURE 2:**
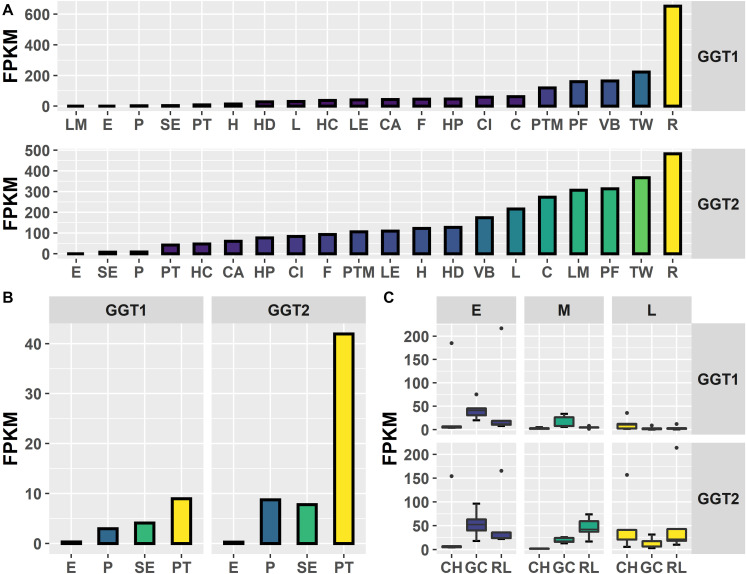
Expression of *JrGGT1* and *JrGGT2*. **(A)** Expression by tissue type of three independent biological replicates pooled together. LM, leaf mature; E, embryo; P, pellicle; SE, somatic embryo; PT, packing tissue; H, hull; HD, hull dehiscing; L, leaf; HC, hull cortex; LE, leaf early; CA, callus exterior; F, fruit; HP, hull peel; CI, callus interior; C, catkin; PTM, packing tissue mature; PF, pistillate flower; VB, vegetative bud; TW, transition wood; R, root. Note that tissue type is ordered by expression level. **(B)** Scaled view of E, P, SE, and PT from **(A)**. **(C)** Expression by genotype and stage. CH, Chandler; GC, Gayle’s Caramel; RL, Robert Livermore; E, early; M, middle; L, late; FPKM, fragments per kilobase of transcript per million mapped reads. Each box and whiskers (genotype/stage) consists of five independent biological replicates.

To investigate *in natura* the relationships between the *JrGGTs* and HBA, HCA, and flavonoid metabolism, we examined RNAs and metabolites associated with *JrGGT1* and *JrGGT2* expression. We used transcriptome data from 20 walnut tissues (T20) (Chakraborty et al., 2016) and transcriptome (Tp) and metabolome (Mp) data of pellicle tissue from three varieties of walnut at three different stages of maturity for our analyses. Tp and Mp data sets were merged according to their respective sample ID to conduct bivariate analysis. Like *UGT84A13*, both *JrGGT*s’ highest expression was in roots, wood, buds, and leaves: tissues that typically accumulate HTs and phenolic acid glycosides (Mittasch et al., 2014) (Figure 2). *JrGGT1* had relatively low expression (<100 FPKM) in most tissues except for in packing tissue mature (PTM), pistillate flower (PF), vegetative bud (VB), transition wood (TW), and root (R) (Figure 2 and Supplementary Table 2). *JrGGT2* was expressed at >100 FPKM in more tissues, including PTM, leaf early (LE), hull (H), hull dehiscing (HD), VB, leaf (L), catkin (C), leaf mature (LM), PF, TW, and R (Supplementary Table 2). Expression of *JrGGT1* in pellicle tissue was about three times less than that of *JrGGT2* in T20 (Figure 2 and Supplementary Table 2). Tp data corroborates that *JrGGT1* expression on average is lower than *JrGGT2* (Figure 2). *JrGGT2* displayed uniquely high expression in leaf tissues compared to *JrGGT1*. Spearman feature correlation analysis resulted in 12,321 RNAs (Tp), 97 metabolites (Mp), and 14,320 RNAs (T20) that correlated significantly with *JrGGT1* expression (Table 2). *JrGGT2* expression correlated with 8,253 RNAs (Tp), 27 metabolites (Mp), and 17,093 RNAs (T20).

**TABLE 2:** Summary statistics of spearman feature correlation analysis of *JrGGT1* and *JrGGT2*.

**Data type**	**Gene**	**Features removed**	**Features analyzed**	**Significant features**	**Positively correlated**	**Negatively correlated**
Tp	*JrGGT1*	9859	33464	12321	7159	5162
Mp	*JrGGT1*	0	209	97	46	51
T20	*JrGGT1*	7523	35800	3875	2447	1428
Tp	*JrGGT2*	9859	33464	8253	4787	3466
Mp	*JrGGT2*	0	209	27	17	10
T20	*JrGGT2*	7523	35800	5749	3424	2325

*Mp, metabolome pellicle; Tp, transcriptome pellicle; T20, transcriptome 20 tissues.*

#### Transcripts Correlated With *JrGGT1*

In T20 and Tp RNA-seq data sets, *JrGGT1* positively correlated with enzymes in the shikimic acid pathway, deoxy-D-arabinoheptulosonate 7-phosphate synthase (*JrDAHPS*) (NCBI 109007614 and 109007613), and shikimate dehydrogenase (*JrSkDH*) (NCBI 109011053 and 109011052) (Figure 1 and Table 3). Both Tp and T20 correlation analyses identified the same *JrDAHPSs* and *JrSkDHs* (Table 3). Genes of the phenylpropanoid pathway also correlated with *JrGGT* expression. *JrGGT1* expression mirrored RNAs encoding phenylalanine ammonia-lyase (*JrPAL*) (NCBI 108997102), bifunctional aspartate aminotransferase and glutamate/aspartate–prephenate aminotransferase (*JrPAT*) (NCBI 109013250), cinnamic acid-4-hydroxylase (*JrCA4H*) (NCBI 108996955), and caffeic acid 3-*O*-methyltransferase (*JrCMT*) (NCBI 109007527 and 109013104). The *JrCMT*s did not correlate with JrGGT1 in T20 (Table 3). *JrGGT1* displayed robust correlations only in the Tp data with expression of leucoanthocyanidin reductase (*JrLAR*) (NCBI 108994581), flavanone 3-hydroxylase (*JrF3H*) (NCBI 108997708), chalcone synthase (*JrCHS*) (NCBI 108995889), dihydroflavonol-4-reductase (*JrDFR*) (NCBI 108991381), and anthocyanidin synthase (*JrANS*) (NCBI 109010746) of the flavonoid biosynthetic pathway (Figure 1 and Table 4).

**TABLE 3:** Selected RNAs and metabolites related to HBA/HCA UGT activity from Spearman feature correlation of *JrGGT1* and *JrGGT2*.

		***JrGGT1***		***JrGGT2***	
**Data set**	**NCBI ID/KEGG compound ID**	**KEGG definition**	***ρ***	**KEGG definition**	***ρ***
Mp	C00493	Shikimic acid ***	0.71	Shikimic acid NS	0.21
Mp	C00296	Quinic acid ***	0.6	Quinic acid NS	0.24
Mp	C00029	(*)UDP-galactose ***	0.51	(*)UDP-galactose NS	0.03
Mp	C00852	Chlorogenate *	0.3	Chlorogenate ***	0.51
Mp	C10788	Ellagic acid **	–0.41	Ellagic acid NS	0.22
Mp	C01197	Caffeate ***	–0.5	Caffeate NS	0.05
Mp	C00628	Gentisate ***	–0.51	Gentisate NS	–0.12
Mp	C06672	Vanillate ***	–0.51	Vanillate NS	–0.18
Mp	C00482	sinapate ***	–0.63	Sinapate NS	–0.22
Tp	109007614	*JrDAHPS* ***	0.78	*JrDAHPS* ***	0.54
Tp	108997102	*JrPAL* ***	0.74	*JrPAL* ***	0.7
Tp	109007613	*JrDAHPS* ***	0.73	*JrDAHPS* ***	0.56
Tp	109007527	*JrCMT* ***	0.72	*JrCMT* ***	0.48
Tp	109013104	*JrCMT* ***	0.72	*JrCMT* **	0.47
Tp	109011053	*JrSkDH* ***	0.7	*JrSkDH* ***	0.66
Tp	109011052	*JrSkDH* ***	0.67	*JrSkDH* ***	0.52
Tp	109013250	*JrPAT* ***	0.61	*JrPAT* *	0.3
Tp	108996955	*JrCA4H* ***	0.57	*JrCA4H* ***	0.57
Tp	109005939	*JrSkDH* **	0.42	*JrSkDH* NS	0.27
T20	109011053	JrSkDH ***	0.85	JrSkDH *	0.53
T20	109011052	JrSkDH ***	0.84	JrSkDH *	0.54
T20	109007614	JrDAHPS ***	0.75	JrDAHPS **	0.6
T20	109007613	JrDAHPS **	0.61	JrDAHPS *	0.52
T20	108996955	JrCA4H *	0.57	JrCA4H *	0.51
T20	109013250	JrPAT *	0.54	JrPAT *	0.49
T20	108997102	JrPAL *	0.51	*JrPAL NS*	0.38
T20	109013104	*JrCMT NS*	0.35	*JrCMT NS*	0.20
T20	109005939	*JrSkDH NS*	0.33	JrSkDH *	0.48
T20	109007527	*JrCMT NS*	0.32	*JrCMT NS*	0.41

Mp, metabolome pellicle; Tp, transcriptome pellicle; T20, transcriptome 20 tissues; *Isobar with UDP-glucose; *ρ*: Spearman rho value (correlation coefficient).

The stars indicate significance by *p*-value.

*p ≤ 0.05, **p ≤ 0.01, ***p ≤ 0.001.

*Significance for Mp and Tp calculated using 45 independent biological replicates.*

*Significance for T20 calculated using twenty independent biological replicates.*

**TABLE 4:** Selected RNAs and metabolites related to flavonoid UGT activity from Spearman feature correlation of *JrGGT1* and *JrGGT2*.

**Type**	**NCBI ID/KEGG compound ID**	**KEGG definition**	***ρ***	**KEGG definition**	***ρ***
Tp	108994581	*JrLAR* **	0.42	*JrLAR* ***	0.84
Tp	108997708	*JrF3H* ***	0.52	*JrF3H* ***	0.77
Tp	108995889	*JrCHS* ***	0.64	*JrCHS* ***	0.72
Tp	108991381	*JrDFR* ***	0.73	*JrDFR* ***	0.71
Tp	109010746	*JrANS* ***	0.52	*JrANS* ***	0.71
Mp		Epicatechin gallate NS	–0.27	Epicatechin gallate *	0.33
Mp		Catechin gallate **	–0.47	Catechin gallate NS	0.18
Mp	C05623	Quercetin 3-*O*-glucoside **	–0.45	Quercetin 3-*O*-glucoside NS	0.13
Mp	C10073	Quercetin 3-galactoside **	–0.45	Quercetin 3-galactoside NS	0.11
Mp	C00389	Quercetin ***	–0.56	Quercetin NS	–0.05
Mp	C09727	Epicatechin **	–0.46	Epicatechin NS	–0.09
Mp	C09099	Naringenin 7-*O*-glucoside ***	–0.51	Naringenin 7-*O*-glucoside NS	–0.13
Mp	C06562	Catechin ***	–0.61	Catechin NS	–0.23
Mp	C00509	Naringenin ***	–0.65	Naringenin NS	–0.28
Mp	C01750	Quercitrin **	–0.44	Quercitrin NS	–0.29
T20	108995889	*JrCHS* NS	0.32	JrCHS *	0.52
T20	109010746	*JrANS* NS	0.31	JrANS *	0.48
T20	108994581	*JrLAR* NS	0.29	JrLAR *	0.47
T20	108991381	*JrDFR* NS	0.22	JrDFR *	0.47
T20	108997708	*JrF3H* NS	0.30	*JrF3H* NS	0.43

*Mp, metabolome pellicle; Tp, transcriptome pellicle; T20, transcriptome 20 tissues; *ρ*, Spearman rho value (correlation coefficient).*

The stars indicate significance by *p*-value.

*p ≤ 0.05, **p ≤ 0.01, ***p ≤ 0.001.

*Significance for Mp and Tp were calculated using 45 independent biological replicates.*

*Significance for T20 was calculated using 20 independent biological replicates.*

#### Metabolites Correlated With *JrGGT1*

1-*O*-Galloyl-β-D-glucose was not detected in Mp, despite being in the compound library. Several phenolic metabolites and precursors involved in HBA/HCA UGT activity correlated strongly with *JrGGT1* expression. Shikimate, QA, UDP-galactose (isobar with UDPG), and chlorogenate correlated positively with *JrGGT1* (Table 3). Many phenolic acids correlated negatively with *JrGGT1*, including HBAs like vanillate, gentisate, and the HCAs caffeic acid and sinapinic acid. The ellagitannin by-product, ellagic acid, also correlated negatively with *JrGGT1*. Surprisingly, the correlation between GA and *JrGGT1* was negative but not significant. This pattern continued into the flavonoids, where *JrGGT1* expression correlated negatively with many flavonoid aglycones and glycones (Table 4). *JrGGT1* did not correlate with epicatechin gallate.

#### Transcripts Correlated With *JrGGT2*

*JrGGT2* correlated less with the shikimic acid pathway enzymes *JrDAHPS*s and *JrSkDH*s than *JrGGT1* in Tp and T20 (Table 3). The phenylpropanoid pathway enzymes *JrPAL*, *JrPAT*, *JrCA4H*, and *JrCMT*s correlated well with *JrGGT2* expression but weaker than *JrGGT1*. *JrPAL* and the two *JrCMTs* did not correlate with *JrGGT2* expression in T20. In Tp and T20, *JrGGT2* correlated better than *JrGGT1* with *JrCHS*, *JrF3H*, *JrDFR*, *JrANS*, and *JrLAR* of flavonoid biosynthesis. All correlations in flavonoid biosynthetic genes were weaker in T20 than in Tp.

#### Metabolites Correlated With *JrGGT2*

A superior correlation was seen between *JrGGT2* and chlorogenate than with *JrGGT1*. However, *JrGGT2* did not correlate with shikimic acid, QA, or UDP-galactose. Moreover, unlike *JrGGT1*, no significant association was seen between *JrGGT2* expression and any of the HBAs, HCAs, and flavonoids, except for a positive correlation with epicatechin gallate (Table 4).

### Expression of *JrGGTs* in Tobacco Plants Altered Metabolites Responsible for HBA, HCA, and Flavonoid Biosynthesis

**FIGURE 3:**
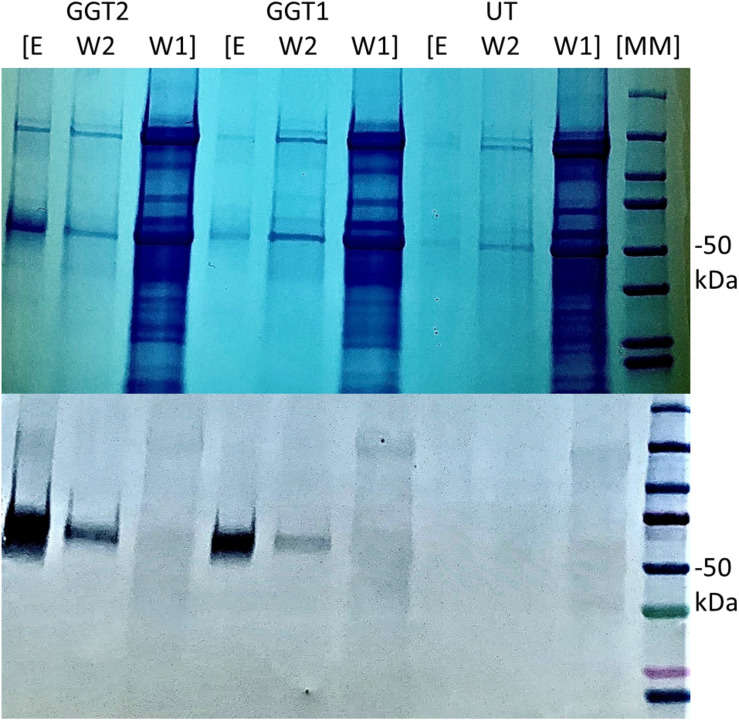
Expression of recombinant *JrGGT1* and *JrGGT2* protein in tobacco leaf tissue. Two gels for SDS-PAGE (top) and Western blot (bottom) were run in tandem and the same samples were used for both. “E, W2, and W1” stand for elution, wash two, and wash one, respectively, from the His-purification process. UT served as an untransformed control. Molecular weight marker (MM) of 50 kDa is denoted by “–50 kDa” on the right side of the figure. Note that the elution step was concentrated using a centrifugal filter and, thus, is an inflated representation of protein expression compared to the other steps of the His-purification process.

We used an anti-his tag antibody for transgenic tobacco screening to select two highest-expressing lines: one for *JrGGT1* and one for *JrGGT2* (Figure 3). Seeds from these T0 plants and untransformed tobacco were germinated on selective and non-selective media, respectively, to create plants grown in the greenhouse. Twelve seedlings, each from untransformed tobacco (UT; grown without kanamycin) and from tobacco plants overexpressing *JrGGT1* and *JrGGT2*, were allowed to grow 4 weeks. Then, leaf tissue was harvested for metabolite and amino acid analysis.

**FIGURE 4:**
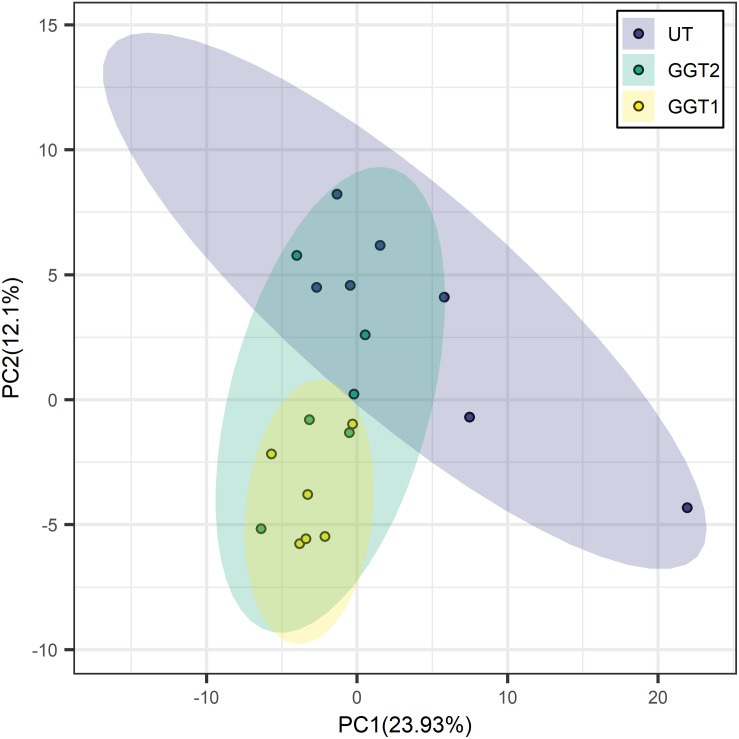
Principal component analysis score clustering of *JrGGT1-* and *JrGGT2*-expressing and UT tobacco lines by GC-MS metabolomics. UT, untransformed. The circles surrounding each class represent 95% confidence intervals. Six independent biological replicates were used for *JrGGT1-* and *JrGGT2*-expressing lines, while seven were used for UT lines.

Transgenic expression of *JrGGTs* in tobacco produced similar metabolic profiles compared to UT. In principal component analysis scores, tobacco lines expressing *JrGGT1* and *JrGGT2* clustered in the same direction and space compared to UT, but with different magnitudes. This result indicates they had similar global metabolomes (Figure 4). *JrGGT1*-expressing samples separated well from UT samples. *JrGGT2-*expressing tobacco lines did not separate well from UT samples or *JrGGT1* samples. Partial least-square-discriminant analysis (PLS-DA) cross-validation performance measures Q2 and accuracy were lower in *JrGGT2-*expressing lines than in *JrGGT1*-expressing tobacco lines. This finding indicates that *JrGGT2*-expressing lines were not as different from UT as *JrGGT1*-expressing lines (Supplementary Figure 3). Compared to UT lines, PLS-DA resulted in 41 metabolites increased and 23 metabolites decreased in *JrGGT1*-expressing tobacco. In *JrGGT2*-expressing lines, this analysis resulted in 44 metabolites increased and 18 metabolites decreased (Table 5). 1-*O*-Galloyl-β-D-glucose was not detected in this study, despite being in the compound library.

**TABLE 5:** Summary statistics of PLS-DA analysis of *JrGGT1-* and *JrGGT2*-expressing tobacco lines.

**Comparison**	**Features removed**	**Features analyzed**	**Significant features**	**Features increased**	**Features decreased**
*JrGGT1*	0	168	64	41	23
*JrGGT2*	0	168	62	44	18

*Features increased means increased in *JrGGT*-expressing lines.*

#### *JrGGT1*-Expressing Lines vs. UT Lines

**FIGURE 5:**
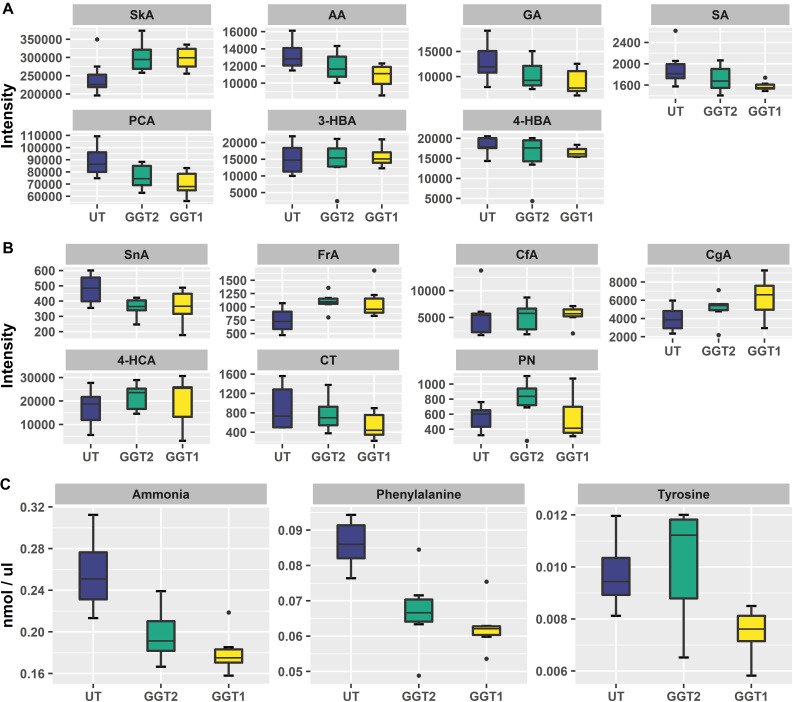
Boxplots of relevant HBAs, HCAs, flavonoids, and amino acids. **(A)** SkA, shikimic acid; AA, *p*-anisic acid; GA, gallic acid; SA, salicylic acid; PCA, protocatechuic acid; 3-HBA, 3-hydroxybenzoic acid; 4-HBA, 4-hydroxybenzoic acid. **(B)** SnA, sinapinic acid; FrA, ferulic acid; CfA, *cis*-caffeic acid; CgA, chlorogenic acid; 4-HCA, 4-hydroxycinnamic acid; CT, catechin; PN, prunin. Six independent biological replicates were used for *JrGGT1-* and *JrGGT2*-expressing lines, while seven were used for UT lines. **(C)** Boxplots of ammonia, phenylalanine, and tyrosine from free amino acid analysis of six independent biological replicates each for *JrGGT1-* and *JrGGT2*-expressing lines and UT lines.

*JrGGT1* tobacco lines had 22% more shikimic acid and 25% more UDP-galactose than UT lines. Only *JrGGT1*-expressing tobacco lines were enriched in chlorogenic acid by 59%. QA was not affected. All differentially expressed HBAs detected were reduced in *JrGGT*-expressing tobacco lines. The HBA plant hormone salicylic acid (SA), the methoxybenzoic acid *p*-anisic acid, 3,4-dihydroxybenzoic acid (PCA), and GA were reduced by 18%, 19%, 21%, and 31%, respectively, in *JrGGT1*-expressing tobacco lines (Table 6 and Figure 5A). Expectedly, benzoic acid remained unchanged. Only *JrGGT1*-expressing tobacco lines had less 4-HBA (p-hydroxybenzoic acid), by 10%. There were no differences in levels of 4-HCA or *cis*-caffeic acid. *JrGGT1-*expressing tobacco lines had 45% more of the sinapinic acid precursor ferulic acid and 24% less sinapinic acid (Figure 5B). Neither line showed significant changes in the HCA *cis*-caffeic acid. Catechin was reduced only in *JrGGT1-*expressing tobacco lines, by 42%. Levels of a flavonoid glycone, 4′,5-dihydroxy-7-glucosyloxyflavanone (prunin), were not changed by *JrGGT1* expression. Only *JrGGT1-*expressing tobacco lines had significantly reduced tyrosine in GC-MS analysis, by 17%. Amino acid analysis of *JrGGT1-*expressing tobacco lines also showed that only tyrosine was reduced by 23% (Figure 5C). Phenylalanine was depleted in *JrGGT1-*expressing tobacco lines by 27% and ammonia reduced by 30%.

**TABLE 6:** Selected metabolites related to HBA/HCA UGT activity from GC-TOF-MS analysis of *JrGGT1-* and *JrGGT2*-expressing tobacco lines.

	***JrGGT1***			***JrGGT2***		
**KEGG compound ID**	**Bin base name**	**FC**	**Log_2_ FC**	**Bin base name**	**FC**	**Log_2_ FC**
C00852	Chlorogenic acid *	1.59	0.67	Chlorogenic acid NS	1.29	0.36
C01494	Ferulic acid *	1.45	0.53	Ferulic acid *	1.46	0.54
C00052	UDP-galactose *	1.25	0.32	UDP-galactose *	1.18	0.24
C00493	Shikimic acid *	1.22	0.28	Shikimic acid *	1.23	0.3
C00811	4-Hydroxycinnamic acid NS	1.17	0.23	4-Hydroxycinnamic acid *	1.28	0.36
C00180	Benzoic acid NS	1.1	0.13	Benzoic acid NS	0.99	–0.02
C00587	3-Hydroxybenzoic acid NS	1.04	0.06	3-Hydroxybenzoic acid NS	0.94	–0.09
C00156	4-Hydroxybenzoate NS	1.04	0.05	4-Hydroxybenzoate NS	1.12	0.16
C01197	*cis*-Caffeic acid NS	1.03	0.04	*cis*-Caffeic acid NS	0.98	–0.03
C09099	4’,5-dihydroxy-7-glucosyloxyflavanone NS	1.01	0.01	4’,5-Dihydroxy-7-glucosyloxyflavanone NS	1.42	0.5
C00296	Quinic acid NS	0.91	–0.13	Quinic acid NS	0.80	–0.33
C00156	4-Hydroxybenzoic acid *	0.90	–0.15	4-Hydroxybenzoic acid NS	0.85	–0.24
C00082	Tyrosine *	0.83	–0.26	Tyrosine NS	1.08	0.11
C00805	Salicylic acid *	0.82	–0.28	Salicylic acid *	0.89	–0.16
C02519	*p*-Anisic acid *	0.81	–0.3	*p*-Anisic acid *	0.9	–0.15
C00230	3,4-Dihydroxybenzoic acid *	0.79	–0.34	3,4-Dihydroxybenzoic acid *	0.85	–0.23
C20448	Sinapinic acid *	0.76	–0.4	Sinapinic acid *	0.75	–0.42
C01424	Gallic acid *	0.69	–0.54	Gallic acid *	0.8	–0.33
C06562	Catechin *	0.58	–0.78	Catechin NS	0.85	–0.23

FC, fold change (average intensity of *JrGGT*OE lines/average intensity of UT lines). The “*” indicates a VIP score greater than the threshold of 1 for significance.

#### *JrGGT2*-Expressing Lines vs. UT Lines

Similar to *JrGGT1*-expressing tobacco lines, *JrGGT2-*expressing lines had 23% more shikimic acid and 18% more UDP-galactose. QA was also unchanged. SA, *p*-anisic acid, PCA, and GA were also reduced by 11%, 10%, 15%, and 20%, respectively, in *JrGGT2*-expressing tobacco lines. Benzoic acid was also not affected. *JrGGT2-*expressing lines showed increases in 4-HCA by 28%, while *cis*-caffeic acid remained unchanged. Like *JrGGT1*-expressing lines, *JrGGT2* expression increased ferulic by 48%, while sinapinic acid decreased by 25%. Catechin remained unchanged; however, *JrGGT2-*expressing lines had 42% more prunin, with a variable importance in projection (VIP) score just below the significance threshold (Figure 5B). *JrGGT2* expression also reduced the amino acid phenylalanine by 22% and ammonia by 27%.

## Discussion

By the convention of the UDP-glycosyltransferase (UGT) Nomenclature Committee (UNC), *JrGGT1* and *JrGGT2* belong to the UGT84A class of enzymes: HBA, HCA, and flavonoid UGTs that derive from the shikimic acid and phenylpropanoid pathways (Lunkenbein et al., 2006; Khater et al., 2012; Mittasch et al., 2014; Cui et al., 2016; Ono et al., 2016). Sequence identity shared by the *JrGGT*s and all functionally characterized UGT84As (Table 1) vastly exceeds 60%, as stated by UNC’s nomenclature guidelines (Mackenzie et al., 2005). Moreover, *JrGGT1*, and to a lesser extent, *JrGGT2*, coexpress tightly with a suite of genes and metabolites of the shikimic acid and phenylpropanoid pathways required for biosynthesis of HBAs, HCAs, and flavonoids (Figure 1 and Tables 3, 4). Non-coincidentally, *JrGGT* expression in tobacco created similar patterns in shikimic acid and phenylpropanoid metabolites. The two transgenic groups produced comparable phenolic and global metabolic profiles (Figures 4, 5 and Tables 6, 7). Together, the genomic, transcriptomic, and metabolomic evidence presented here suggests that *JrGGT1* and *JrGGT2* play important roles in the metabolism of HBAs, HCAs, and flavonoids.

**TABLE 7:** Tyrosine, phenylalanine, and ammonia from free amino acid analysis of *JrGGT1-* and *JrGGT2*-expressing SR1 lines.

***JrGGT1***			***JrGGT2***		
**Biochemical**	**FC**	**Log_2_ FC**	**Biochemical**	**FC**	**Log_2_ FC**
Tyrosine	0.77	−0.38	Tyrosine	1.04	0.06
Phenylalanine	0.73	−0.46	Phenylalanine	0.78	–0.36
Ammonia	0.70	−0.51	Ammonia	0.77	–0.38

*Fold change is the average intensity of *JrGGT1* lines/average intensity of UT lines. Comparison is to UT.*

As the UGT84As tend to show high affinity toward GA and PCA *in vitro*, it was not surprising to see that *JrGGT1* and *JrGGT2* positively correlated with the *JrDAHPSs* and *JrSkDHs in vivo* (Khater et al., 2012; Mittasch et al., 2014; Ono et al., 2016) (Figure 1 and Table 3). *JrGGT1*, in particular, showed robust positive correlations with two *JrDAHPSs*, while *JrGGT2*’s correlations with these genes were weaker. *JrGGT2* also did not correlate as strongly with the *JrSkDHs* (Table 3). Perhaps these results indicate that the two *JrGGTs* have different biochemical functions. The unique relationship between the *JrGGT*s, *JrDAHPSs*, and *JrSkDHs* in Table 3 may indicate they evolved to work together, as many *JrDAHPSs* and *JrSkDHs* did not correlate with the *JrGGTs*. Both *JrGGT*s’ correlations with *JrSkDH* (NCBI 109005939) were far weaker than that of the *JrSkDHs* (NCBI 109011053 and 109011052). This finding was intriguing, given that *JrSkDH* (NCBI 109005939) was described as responsible for GA biosynthesis in *Juglans regia* (Muir et al., 2011). The strong positive correlations between *JrGGT1* expression, shikimic acid, and QA corroborate the relationships between *JrGGT1*, the *JrDAHPSs*, the *JrSkDHs*, and thus, the shikimic acid trunk (Figure 1 and Table 3). The lack of correlations between *JrGGT2* expression, shikimic acid, and QA is somewhat consistent with this gene’s weaker association with the *JrDAHPS* variants and the *JrSkDHs* compared to *JrGGT1*. Despite the correlation results, *JrGGT2-*expressing tobacco lines accumulated more shikimic acid than UT (Table 6). *JrGGT1-*expressing tobacco lines also accumulated more shikimic acid, contributing to our hypothesis that these genes are involved in shikimic acid metabolism (Figure 5A and Table 6). These findings were interesting, given that we predicted that *JrGGT* overexpression would deplete free shikimic acid. In a similar case of confusion, *SkDH* silencing in tobacco increased concentrations of the substrate (3-DHQ) and its product, shikimic acid (Ding et al., 2007). Since the SkDHs are NADP+/(H) dependent, it is possible that altered metabolism along the shikimic acid pathway could shift the NADP+: NADPH ratio near the enzyme. Redox homeostasis could regulate the shikimic acid concentration, with NADP+ favoring oxidation to GA and NADPH favoring reduction to shikimic acid (Figure 1). Similarly, cofactor balance may necessitate that any additional carbon flowing toward the GA pathway would require a commensurate amount of carbon to flux toward shikimic acid biosynthesis. The generation of GA can result in NADPH production (Figure 1), which may then be used to facilitate reduction of shikimic acid and subsequent generation of NADP+, which can again drive the oxidation of 3-DHS to GA, forming a small cycle. Put shortly, GA and shikimic acid biosynthesis may be inexorably linked, as suggested previously (Muir et al., 2011). Similar to the shikimic acid results, *JrGGT1* correlated strongly with UDP-galactose, and its expression in tobacco resulted in accumulation of UDP-galactose, indicating that it is also involved in UDP sugar metabolism (Table 3). *JrGGT2* expression did not correlate with UDP-galactose, yet *JrGGT2*-expressing lines accumulated more UDP-galactose than UT. Perhaps *JrGGT2* is less active than *JrGGT1*.

Evidence that *JrGGT1* regulates HBAs was provided by the negative correlation between *JrGGT1* expression and the HBAs vanillate and gentisate (Table 3). This negative relationship between enzyme and substrate is sensible if JrGGT1 is responsible for glycosylation of vanillate and gentisate. Increased expression of HBA-glycosylating enzymes (JrGGT1) would reduce aglycones of vanillate and gentisate, while decreased expression of HBA-glycosylating enzymes would favor their increase. Echoing JrGGT2’s effects in tobacco, *JrGGT2* expression also correlated negatively with vanillate and gentisate. However, this relationship was not significant (Figure 5A and Tables 3, 6). Indeed, JrGGT orthologs have high substrate affinity with vanillate (Lunkenbein et al., 2006; Mittasch et al., 2014). Vanillate could be a preferred HBA for JrGGT1 in pellicle tissue. Surprisingly, *JrGGT* expression did not correlate significantly with GA in pellicle metabolite data, despite GA being a famous metabolite in the field of UGT84A research. This finding may indicate that GA is not a principal substrate for the JrGGTs *in natura*. *JrGGT* overexpression in tobacco either reduced or did not significantly change the relative abundance of all HBAs, supporting the correlations with HBAs in Mp (Figure 5A and Table 6). The most significantly reduced HBAs in both lines were GA, PCA, and the plant hormone SA. Surprisingly, the effects of *JrGGT* expression on GA were inconsistent with the correlation data, given that GA did not correlate with *JrGGT* expression. Perhaps this result was somehow confounded by *JrGGT* expression in tobacco and not walnut. Also reduced was 4-HBA, but this was only significant in *JrGGT1*-expressing lines. In both lines, the order of magnitude of reduction was GA>*PCA*>*SA*>4-HBA (Table 6). Intriguingly, this echoes similarity with the order-specific activity of VvGT1-3 for PCA and 4-HBA, but not that of UGT84A23-24, and suggests a preference for GA over PCA, and for PCA over SA and 4-HBA *in vivo* when expressed in tobacco (Khater et al., 2012; Ono et al., 2016).

Both *JrGGT1* and *JrGGT2* expression mirrored that of *JrPAL* (Table 3). Coupled with this observation is the fact that *JrGGT1-*expressing tobacco lines showed reductions in tyrosine, phenylalanine, and ammonia, respectively, while in *JrGGT2*-expressing tobacco lines, only phenylalanine and ammonia were reduced (Figure 5C and Table 7). This finding suggests that the JrGGTs are intimately connected with the PAL reaction. Both *JrGGT*s correlated strongly with *JrCA4H* and *JrCMT* RNAs in Tp data (Table 3). However, these relationships tended to be weaker in *JrGGT2* than in *JrGGT1*. *JrGGT1* also correlated negatively with the HCAs caffeate and sinapate (also sinapinic acid), while this relationship with *JrGGT2* was not significant (Table 3). Associations with *JrGGT* expression and genes and metabolites of the phenylpropanoid pathway were verified to be causal in metabolomics of *JrGGT*-expressing tobacco. 4-HCA and ferulic acid were enriched, caffeic acid was unchanged, and sinapinic acid was reduced in *JrGGT1*- and *JrGGT2*-expressing tobacco lines (Figure 5B and Table 6). The reductions in sinapinic acid suggest that it may be the preferred HCA for JrGGT1 and JrGGT2 activity *in vivo*. Indeed, sinapinic acid concentration and *JrGGT1* expression also had a negative relationship in Mp data (Table 3). Moreover, sinapinic acid was glycosylated by UGTs from strawberry, pomegranate, tea, and grape (Lunkenbein et al., 2006; Khater et al., 2012; Cui et al., 2016; Ono et al., 2016). The reductions in preceding metabolites phenylalanine and ammonia, increases in 4-HCA and ferulic acid, and reductions in sinapinic acid could indicate some form of feedback regulation at PAL, as in the phenylpropanoid pathway (Blount et al., 2000).

In contrast to the shikimic acid and phenylpropanoid pathways, *JrGGT2* correlated better with genes and metabolites of flavonoid metabolism than *JrGGT1*. Both *JrGGT*s displayed similar correlations with *JrCHS* and *JrDFR* in Tp; however, this similarity diminished in T20, with *JrGGT2* alone retaining significance (Table 4). The relationships between *JrF3H*, *JrLAR*, *JrANS*, and *JrGGT2* were highly robust in Tp data, while that of *JrGGT1* was less compelling. This difference may indicate that *JrGGT2* is more involved in the metabolism of flavanols, leucoanthocyanidins, flavan-3-ols, and anthocyanidins. Indeed, *JrGGT2* showed a positive relationship with the flavan-3-ol epicatechin gallate, while *JrGGT1* showed no correlation with these flavonoids (Table 4). Biosynthesis of epicatechin gallate in tea requires the HBA glucoside 1-*O*-galloyl-β-D-glucose to donate a galloyl moiety in a galloyltransferase reaction (Liu et al., 2012). *JrGGT2* may be involved in supporting the available pool of 1-*O*-galloyl-β-D-glucose required to synthesize epicatechin gallate. Moreover, *JrGGT2*-expressing, but not *JrGGT1*-expressing, tobacco lines had 42% more of the flavanone glucoside prunin, with a VIP score just below the threshold (Table 6). Similarly, only *JrGGT2-*expressing tobacco lines had 28% more of the flavonoid precursor 4-HCA (Table 6). Perhaps this was to provide more carbon to serve the pathway through CHS. Alternatively, it was *JrGGT1*-expressing and not *JrGGT2*-expressing tobacco lines that were reduced in the flavan-3-ol catechin (Table 6). The reduction of catechin in *JrGGT2*-expressing tobacco lines was not significant, which is curious as *JrGGT2* correlated strongly with *JrLAR* in the Tp and T20 data sets, while *JrGGT1* was not so convincing (Table 4). Confirming this observation was that the catechin fluctuations correlated negatively with *JrGGT1* expression, and not with *JrGGT2* expression.

## Conclusion

Experimental evidence from genomic, transcriptomic, and metabolomic perspectives suggests the JrGGTs are UDP84A-type glycosyltransferases and are involved in shikimic acid and phenylpropanoid metabolism of phenolic acids and flavonoids. While JrGGT1 and JrGGT2 share high sequence similarity at the nucleotide and amino acid levels, their correlations within plant secondary metabolism and gene expression appear different. Considering the evidence provided by Tp, Mp, T20, and *JrGGT* overexpressing tobacco lines, JrGGT1 seems more involved in the metabolism of HBAs, HCAs, and some flavonoids than JrGGT2. Alternatively, these results suggest that JrGGT2 may be more involved in the metabolism of specific flavonoids. Further adding to this point are the differential expression profiles of *JrGGT1* and *JrGGT2* in the T20 expression data.

This study focused on the smaller molecular weight compounds detectable by GC-MS methods and deepened our understanding of JrGGT1 and JrGGT2 and their unique roles in plant secondary metabolism. However, only one phenol glycoside was detected in GC-MS profiling of *JrGGT*-expressing tobacco. Due to their greater molecular weight, tannins and phenolic glycosides are typically not detectable using a GC-MS platform. However, 1-*O*-galloyl-β-D-glucose was detectable yet not observed in the GC-MS tobacco metabolomics. Interestingly, the Mp data generated using a combination of LC-MS and GC-MS techniques also detected no HBA glycosides, despite 1-*O*-galloyl-β-D-glucose being detectable. Therefore, an untargeted LC-MS approach focused on polyphenolics may shed light on the potential effects of *JrGGT* expression on the HTs, tea tannins, and phenolic glycosides *in vivo*. Moreover, the GC-MS tobacco data lacked an empty vector control. These results would be strengthened by adding a PDH17 vector lacking *JrGGT* CDS to provide additional confidence that these effects were not due to transgene insertion.

## Data Availability Statement

Raw metabolite data for of *JrGGT1* and *JrGGT2* overexpressing tobacco were deposited at https://www.ebi.ac.uk/metabolights/search as study MTBLS2042. The raw reads for the RNA-seq analysis of walnut pellicle tissue were deposited to the Sequence Read Archive, https://www.ncbi.nlm.nih.gov/sra, under the BioProject ID: PRJNA663110. Detailed information on the walnut pellicle data can be found in a dissertation (Butterfield, 2017).

## Author Contributions

HS and AD conceived and designed the experiments. HS coordinated and performed the experiments and functional analysis, and wrote the manuscript, which was then edited by TB, TH, NF, BB, and AD. TH performed cloning experiments. TB and BB acquired and processed pellicle RNA-seq and metabolite data. NF and CZ performed the experiments and helped with data collection. AJ maintained plant material. AD and all other authors revised the final manuscript. All authors contributed to the article and approved the submitted version.

## Conflict of Interest

The authors declare that the research was conducted in the absence of any commercial or financial relationships that could be construed as a potential conflict of interest.

## Footnotes

1 https://ptf.ucdavis.edu/

2 http://www.bioinformatics.babraham.ac.uk/projects/fastqc/

3 http://www.arb-silva.de

4 ftp://ftp.ncbi.nlm.nih.gov/genomes/all/GCF/001/411/555/GCF_001411555.1_wgs.5d/GCF_001411555.1_wgs.5d_genomic.fna.gz

## Supplementary Material

Supplementary Figure 1 Schematic of binary vectors pDH17.0301 and pDH17.0401, used to express *JrGGT1* and *JrGGT2*, respectively, in transgenic *Nicotiana tabacum*.

Supplementary Figure 2 Schematic of bioinformatics of RNA-seq data.

Supplementary Figure 3 Cross-validation of **(A)** *JrGGT1* and **(B)** *JrGGT2* PLS-DA, displaying three model performance measures: the sum of squares captured by the model (R2), the cross-validated R2 (also known as Q2), and the prediction accuracy (Accuracy). The default criterion is Q2 (marked by a red star), which indicates the optimal number of components to use during modeling.

Supplementary Table 1 Primers used in cloning and vector construction.

Supplementary Table 2 Expression of *JrGGT1* and *JrGGT2* across 20 tissues in fragments per kilobase per million mapped reads (FPKM) of walnut.

## Abbreviations

3-DHQ: 3-dehydroquinate
3-DHS: 3-dehydroshikimate
AA: *p*-anisic acid
ADT: arogenate dehydratase/prephenate dehydratase
ANS: leucoanthocyanidin dioxygenase, anthocyanidin synthase
CA4H: cinnamic acid-4-hydroxylase
CHS: chalcone synthase
CMT: caffeic acid 3-*O*-methyltransferase
CS: chorismate synthase
CSE: caffeoylshikimate esterase
DAHPS: 3-deoxy-D-arabinoheptulosonate 7-phosphate synthase
DFR: dihydroflavonol-4-reductase
DHQS: dehydroquinate synthase
E-4-P: erythrose-4-phosphate
F3H: naringenin2-oxoglutarate 3-dioxygenase flavanone 3-hydroxylase
F5H: ferulate-5-hydroxylase
GA: gallic acid
GGT: galloyl glycosyltransferase
HBA: hydroxybenzoic acid
HCA: hydroxycinnamic acid
HCT: hydroxycinnamoyl-CoA: shikimate/quinate hydroxycinnamoyl transferase
LAR: leucoanthocyanidin reductase
Mp: metabolome pellicle
PAL: phenylalanine ammonia lyase
PAT: bifunctional aspartate aminotransferase and glutamate/aspartate-prephenate aminotransferase
PCA: protocatechuic acid, 3,4-dihydroxybenzoic acid
PEP: phosphoenolpyruvate
QA: quinic acid
SA: salicylic acid
SkA: shikimic acid
SkDH: bifunctional dehydroquinate dehydratase/shikimate dehydrogenase
T20: transcriptome 20 tissues
Tp: transcriptome pellicle
UDPG: UDP-glucose
UGT: uridine diphosphate glycosyltransferase
UNC: UGT Nomenclature Committee.
