# Supplementary material for: Two UGT84A Family Glycosyltransferases Regulate Phenol, Flavonoid, and Tannin Metabolism in Juglans regia (English Walnut)
Source: Front Plant Sci. 2021 Feb 24;12:626483. doi: 10.3389/fpls.2021.626483 (PMC7943615; doi:10.3389/fpls.2021.626483)
Supplement: Supplementary Figure 1 — Schematic of binary vectors pDH17.0301 and pDH17.0401, used to express JrGGT1 and JrGGT2, respectively, in transgenic Nicotiana tabacum. [file Data_Sheet_1.docx]

Two UGT84A Family Glycosyltransferases Regulate Phenol, Flavonoid and Tannin Metabolism in Juglans regia (English Walnut)

Supplementary Material


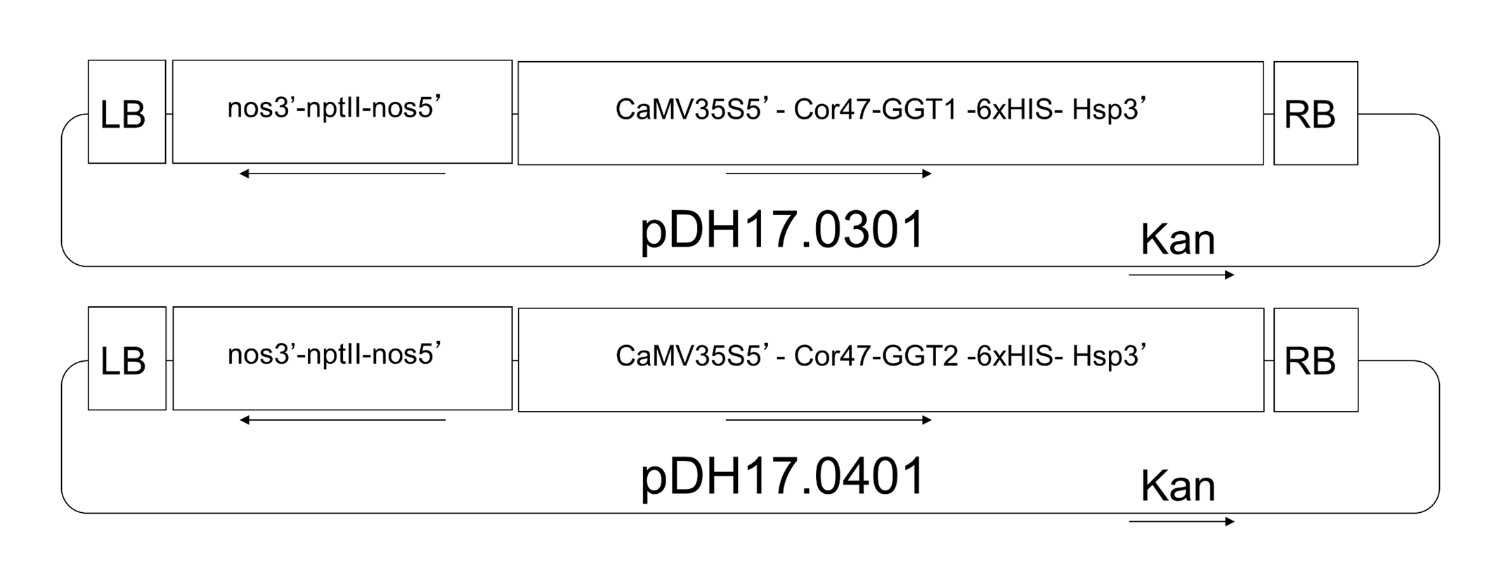


**Figure S1.** Schematic of binary vectors pDH17.0301 and pDH17.0401, used to express *JrGGT1* and *JrGGT2,* respectively, in transgenic *Nicotiana tabacum*.


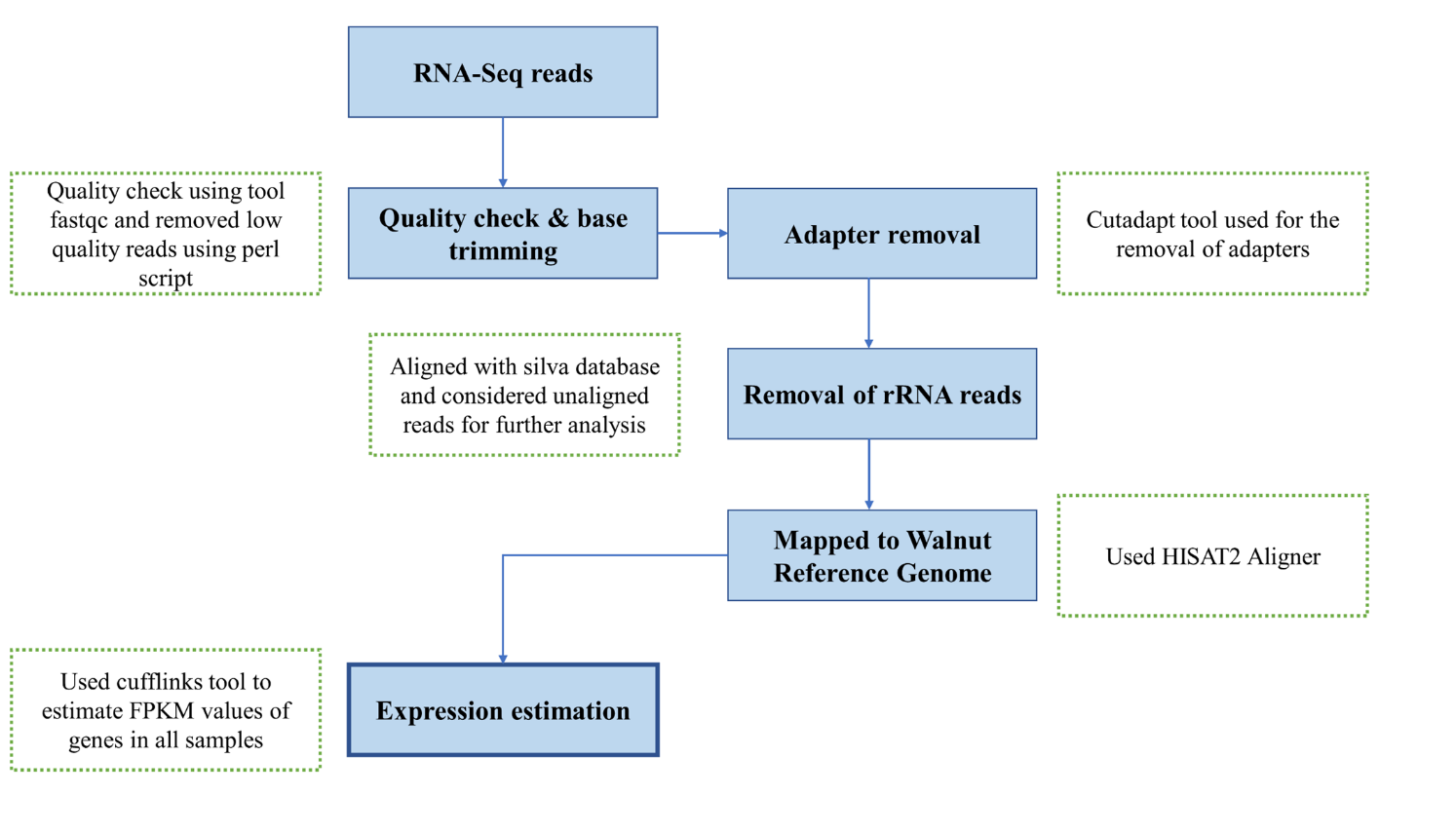


**Figure S2**. Schematic of bioinformatics of RNA-seq data


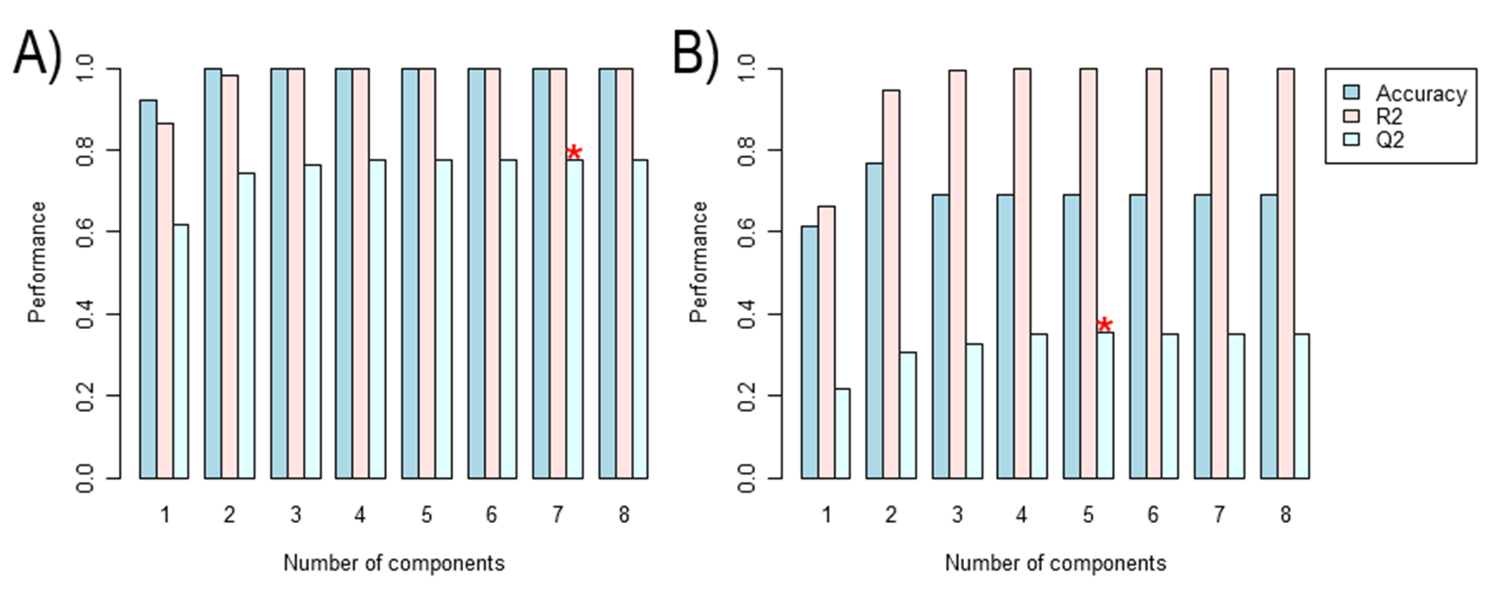


**Figure S3.** Cross-validation of **A)** *JrGGT1* and **B)** *JrGGT2* PLS-DA, displaying three model performance measures: the sum of squares captured by the model (R2), the cross-validated R2 (also known as Q2), and the prediction accuracy (Accuracy). The default criterion is Q2 (marked by a red star), which indicates the optimal number of components to use during modeling.

| **Table S1.** Primers used in cloning and vector construction | | |
| --- | --- | --- |
| **Purpose** | **Forward** | **Reverse** |
| Cloning for *JrGGT1* | ATGGGATCCGAAGCTCTAGTCCA | TTAGGATGCAACCAGGTTCAG |
| Cloning for *JrGGT2* | ATGGGATCTGAAGCTGTGGTCC | TTAGGATACAACCAAGTTGGCGTTCC |
| **pDH17.0301 (*JrGGT1*OE)** | | |
| Linearized cloning vector | GAGCTCATATGAAGATGAAGATGAAATATTTGGTGT | CTTTAATCTTGATTTGATTAAAAGTTTATATAGTAAATAGAAAATATGA |
| Fragment A | AAATCAAGATTAAAGATGGGATCCGAAGCTCTAGTCC | TCTTCATATGAGCTCTTAGTGGTGGTGGTGGTGGTGGC |
| **pDH17.0401 (*JrGGT2*OE)** | | |
| Linearized cloning vector | GAGCTCATATGAAGATGAAGATGAAATATTTGGTGT | CTTTAATCTTGATTTGATTAAAAGTTTATATAGTAAATAGAAAATATGA |
| Fragment B | AAATCAAGATTAAAGATGGGATCTGAAGCTGTGGT | TCTTCATATGAGCTCTTAGTGGTGGTGGTGGTGGTGGC |

| **Table S2.** Expression of *JrGGT1* and *JrGGT2* across 20 tissues of walnut in fragments per kilobase per million mapped reads (FPKM) | | | |
| --- | --- | --- | --- |
| Tissue* | *JrGGT1* | Tissue* | *JrGGT2* |
| LM | 0.00 | E | 0.27 |
| E | 0.30 | SE | 7.78 |
| P | 2.97 | P | 8.76 |
| SE | 4.12 | PT | 41.97 |
| PT | 8.95 | HC | 47.88 |
| H | 14.56 | CA | 60.26 |
| HD | 28.19 | HP | 76.96 |
| L | 30.86 | CI | 83.82 |
| HC | 38.07 | F | 92.75 |
| LE | 41.00 | PTM | 105.64 |
| CA | 43.31 | LE | 109.63 |
| F | 46.15 | H | 122.04 |
| HP | 46.52 | HD | 127.68 |
| CI | 58.43 | VB | 174.43 |
| C | 61.77 | L | 216.14 |
| PTM | 119.63 | C | 273.47 |
| PF | 159.98 | LM | 307.27 |
| VB | 164.26 | PF | 313.72 |
| TW | 222.58 | TW | 367.21 |
| R | 652.11 | R | 482.73 |

*LM: leaf mature; E: embryo; P: pellicle; SE: somatic embryo; PT: packing tissue; H: hull; HD: hull dehiscing; L: leaf; HC: hull cortex; LE: leaf early; CA: callus exterior; F: fruit; HP: hull peel; CI: callus interior; C: catkin; PTM: packing tissue mature; PF: pistillate flower; VB: vegetative bud; TW: transition wood; and R: root.
